# Pulmonary Tuberculosis

**Published:** 1894-04-14

**Authors:** 


### Medical Progress.

### PULMONARY TUBERCULOSIS.

*Cause of Hectic.*—Pyogenic cocci[1] were found in the blood of phthisical patients in a hectic state, thus confirming the generally accepted view that the hectic fever of phthisical patients is due to the presence in the circulation of the products of pyogenic organisms. The blood was taken from the finger in nine patients, and in seven the micro-organisms were detected, *e.g.*, staphylococcus pyogenes aureus, staphylococcus pyogenes albus, streptococcus pyogenes.

Can we induce a temporary or lasting immunity against tuberculosis by inoculation? Richet[2] recently exhibited several dogs successfully vaccinated against tuberculosis. One of these was inoculated on December 6th, 1892, with aviary tuberculosis filtered through paper. In May, together with five unvaccinated dogs, it was inoculated with human tuberculosis. All five unvaccinated dogs died within four months, the vaccinated dog remained healthy, and continued to be so after nine months had elapsed.

Similar experiments were carried out with the other dogs, in each case the five unvaccinated dogs died, while the previously vaccinated ones remained healthy.

Unfortunately, such vaccination was not without danger, inasmuch as from 50 to 75 per cent. of the animals died from the effects of the vaccination.

Dr. Gilbert, in the course of the discussion following Richet's remarks, said that he had endeavoured in vain to vaccinate guinea-pigs against human tuberculosis by inoculating first with aviary tuberculosis, and Richet himself reminded his audience that Grancher and H. Martin had also failed with guinea-pigs.

*Clinical and Pathological Phenomena.*—Two cases of acute phthisis following destruction of the mucous membrane of the stomach by corrosive fluids were brought before the Clinical Society (January 12th) by Dr. Soltau Fenwick.[3] It appeared to him that the sudden impairment of nutrition had so diminished the resistance of the tissues as to render them peculiarly vulnerable to tuberculous invasion.

Professor Delépine[4] recently advanced some views on a certain mode of spread of tuberculosis through the lymphatics.

*Toxæmia in Tuberculosis* is referred to in reporting a case, by Wm. Osler,[5] who states that the symptoms of profound intoxication in tuberculosis are met with under three conditions—(*a*) In those rare cases described most commonly in children, in which death may occur with symptoms of a profound toxæmia before there are any extensive localised foci of disease; (*b*) acute miliary tuberculosis is often accompanied with toxic features, giving to many of the cases clinical pictures of severe typhoid fever. P.m., miliary tubercles are found extensively throughout the viscera and on the serous surfaces. (*c*) In chronic pulmonary tuberculosis these may develop, with or without fever, a profound toxæmia, with dry tongue, delirium, rapid pulse, and signs of intense intoxication.

The relation of febrile condition of phthisis to the prognosis is discussed by Dr. Strümpel.[6] As to the cause of the fever, he is of opinion that, except in miliary cases, it is not to be ascribed to the tuberculosis, but to secondary inflammatory processes set up by the invasion of pus and other cocci, while in a number of cases there is no fever at all. The presence or absence of fever in tubercular phthisis is referred to also by Dr. G. Hunter Mackenzie,[7] who states that "whilst these bacilli (in the sputum) are frequently accompanied by fever—the well-known pyrexia of phthisis—in other instances, even when fairly numerous in the expectoration, the temperature of their hosts is normal for more or less lengthened periods. In the latter class of cases the mercury may now and again mark a slight increase of body heat, but this may not extend over more than a day or two at a time, and is not necessarily accompanied or followed by any appreciable general effect upon the individual. It thus happens that some non-febrile patients in whose expectoration bacilli were found seven, eight, and nine years ago are still alive, and fairly well. They are, however, probably more subject to colds and 'catarrhs' than their healthy fellows. Whilst the course of these chronic bacillary cases may be, on the whole, fairly favourable, it is not always so. A short and sharp accession of the disease may occur, followed by fatal collapse." Such cases would be properly classed in the third type of toxæmic cases referred to by Osler.

Strümpel[8] classifies the various forms of fever in phthisis as follows: 1. *Status subfebrilis* (morning, normal; evening, 100·4° to 101·3° F.) In such cases the disease makes but slow progress, and amelioration may be expected by improving the general condition. 2. *Febris hectica intermittens.* With this type of fever (morning, nearly normal; evening, 101·3° to 104° F.) the disease is steadily advancing, though in such cases a feeling of good health may be maintained for a considerable time. 3. *Febris remittens* (morning, 100·4° to 101·3° F.; evening, 103·1° F). This type is far more unfavourable, as it points to the presence of lobular inflammation. 4. *Febris continua.* Except in miliary tuberculosis, this form of fever is found almost exclusively in cases of phthisis which start with acute symptoms, though it may be found interpolated for several days in cases in which the fever otherwise runs a remittent or irregular course. In either case the prognosis is unfavourable. That totally irregular form which is observed throughout the whole illness in many cases, though only in the last stage in others. Granted that the fever forms a most important indication in prognosis, it follows that it must also be the standard by which antiphthisical remedies must be tested, for it is only by their effect on the fever that a just estimate of their value can be arrived at. On these grounds Strümpell rejected both tuberculin and creosote in the treatment of phthisis: the former he considers to be absolutely injurious, having frequently seen apyretic converted into febrile forms by injections of Koch's specific.

The value of supraclavicular inspiratory depression as a clinical sign of early phthisis when the other indications are slight and doubtful is once more emphasised by Dr. Revillet.[9] The depression is due to the existence of apical pleurisy.

The cause, course, and treatment of a group of non-tubercular cases is discussed by Dr. H. S. Straight[10] Cleveland, O.).

Dr. Osler's Shattuck lecture for 1893 was on tuberculous pleurisy. Of 101 cases of pleurisy examined post-mortem thirty-two were definitely tubercular, and thirteen existed in patients with tubercular lesions of the lungs without any definite proof of the tubercular character of the pleurisy. Osler recognises three distinct clinical types—(1) Acute tubercular pleurisy, rarely fatal, often completely recovering; (2) subacute and chronic forms; (3) general serous membrane tuberculosis, often very chronic and apyretic.

*Prophylaxis and Hygiene.*—Among the more important contributions must be reckoned Dr. G. E. Squire's work on "The Hygienic Prevention of Consumption." Under the head of State Hygiene he remarks that although in Great Britain no laws have been specially enacted with the view of preventing consumption, yet the general improvement in sanitary matters has had the result of reducing the number of deaths from phthisis per million from 54,231 in 1870 to 48,366 in 1890; this represents a saving of about 30,000 lives per annum.

The following remarks by Dr. Sinclair Coghill in the course of his annual report of the Royal National Hospital for Consumption and Diseases of the Chest emphasise the importance of prophylactic measures: "There is now no doubt that in those instances in which several members of one family successively succumbed to consumption, and which occasioned the wide assumption of the hereditary nature of the disease, the fatal results were due not to inheritance but really to infection. It is now ascertained beyond question that the contagious material is contained in the sputum of the affected persons, and that this when dried becomes the vehicle of infection."

All vessels and handkerchiefs are carefully disinfected after use, and instructions are given regarding the expectoration to all the patients.

*Sterilisation of Milk.*—The effect of high temperatures on the tubercle bacillus has been investigated by De Man.[11] He found that tubercle bacilli in milk are destroyed by heating it to 55° C. after four hours, to 60° C. after one hour, to 65° after a quarter of an hour, or by heating to 70° for ten minutes, and by raising the temperature to 90° or 100° in two and one minute respectively. De Man found that heating milk to 70° C. for ten minutes did not alter its taste, while the tubercle bacilli were invariably destroyed.

*Tubercle Bacilli in Cigars.*—The influence of tobacco on tubercle bacilli has been investigated by Dr. Kerez,[12] and he came to the conclusion that tobacco exercises a destructive action on tubercle bacilli, and that a tubercular infection through cigars prepared by phthisical workers is practically impossible.

*Tubercle Bacilli in House and Hospital Ward Dust.*—The dry dust from a house in which there had been a series of cases of phthisis was examined by R. S. Miller.[13] After giving the instructive history of the house in relation to cases of phthisis arising within it, he found numerous tubercle bacilli in the dust scraped off the top of the dining-room door and other places.

The same topic in relation to hospital dust has been investigated by Heron and Chaplin,[14] and their experiments tended to prove that the precautions adopted in the hospital in regard to the use of disinfecting spitoons, &c., were efficient means in preventing the accumulation of bacilli in places unexposed to the destroying power of sunlight.

The experiments of Ransome and Delépine,[15] to test the value of the methods used by the municipality of Manchester for disinfecting rooms which had been occupied by tuberculous persons, seem to show that wall paper infected with bacilli exposed to sunlight for forty-five days were non-infective, while similar paper dried in the dark, or exposed to the action of euchlorine in rooms that were being disinfected, were infective, and that the bacilli were therefore not destroyed by the action of this method of disinfection.

[1] Cent. f. Bakt., Dec. 9, 1893. [2] Med. Week, Feb. 23, 1894. [3] Lancet, Jan. 20, 1894. [4] Manch. Path. Soc.; Lancet, Mar. 10, 1894. [5] Pract., Jan. 25, 1894. [6] Muench. Med. Woch.; Med. Rec., Feb. 3, 1894. [7] Brit. Med. Journ., Mar. 3, 1894. [8] Coccit. [9] Med. Week. [10] Med. Rec., Jan. 6, 1894. [11] Arch. f. Mgz., xviii., p. 134. [12] Scheizer Aerzte Zurich, Jan. 1, 1894; Prov. Med. Journ., Feb., 1894. [13] Brit. Med. Journ., Jan. 6, 1894. [14] Lancet, Jan., 1894. [15] Brit. Med. Journ., 1893, p. 1,714.

(*To be continued.*)

